# Hodgkin Reed–Sternberg‐Like Cells in T‐Cell/Histiocyte‐Rich Large B‐Cell Lymphoma

**DOI:** 10.1002/jha2.70392

**Published:** 2026-09-23

**Authors:** Ulysses Mustaki, Stephen Lade, Mary Ann Anderson, Kah Lok Chan

**Affiliations:** ^1^ Department of Pathology Peter MacCallum Cancer Centre Melbourne Victoria Australia; ^2^ Department of Clinical Haematology The Royal Melbourne Hospital and Peter MacCallum Cancer Centre Melbourne Victoria Australia; ^3^ Division of Blood Cells and Blood Cancer The Walter and Eliza Hall Institute Melbourne Victoria Australia; ^4^ Department of Medical Biology The University of Melbourne Melbourne Victoria Australia; ^5^ Sir Peter MacCallum Department of Oncology The University of Melbourne Melbourne Victoria Australia

**Keywords:** Hodgkin Reed–Sternberg‐like, HRS‐like, T‐cell/histiocyte‐rich large B‐cell lymphoma, THRLBCL

1

A 54‐year‐old man was referred with a 2‐month history of increasing left‐sided cervical lymphadenopathy and lethargy. Peripheral blood counts were normal, but lactate dehydrogenase was significantly elevated (963 U/L), and a staging PET scan showed generalised, intensely FDG‐avid lymphadenopathy and multifocal extranodal disease involving the lung, left flank and bones. Bone marrow biopsy showed extensive infiltration by a polymorphous lymphohistiocytic infiltrate containing admixed large cells (Figure 1A), including some Hodgkin Reed–Sternberg (HRS)‐like binucleate cells with prominent nucleoli (Figure 1B). Immunohistochemistry revealed that the large cells were positive for CD20, PAX5, MUM1 and OCT2 (Figure 1C–F) and negative for CD15, CD30 and EBV‐encoded RNA (Figure 1G–I). A diagnosis of T‐cell/histiocyte‐rich large B‐cell lymphoma (THRLBCL) was made, with targeted DNA sequencing demonstrating a supportive molecular profile including *JUNB*, *CARD11*, *FAS, PLCG2* and *STAT3* variants, as well as aberrant somatic hypermutation of *CIITA*, *DUSP2*, *PAX5* and *SOCS1*. Cervical lymph node biopsy also showed a population of large, polylobated B cells displaying lambda immunoglobulin light chain restriction and a concordant immunophenotype, within a polytypic T‐cell‐rich background.

**FIGURE 1:**
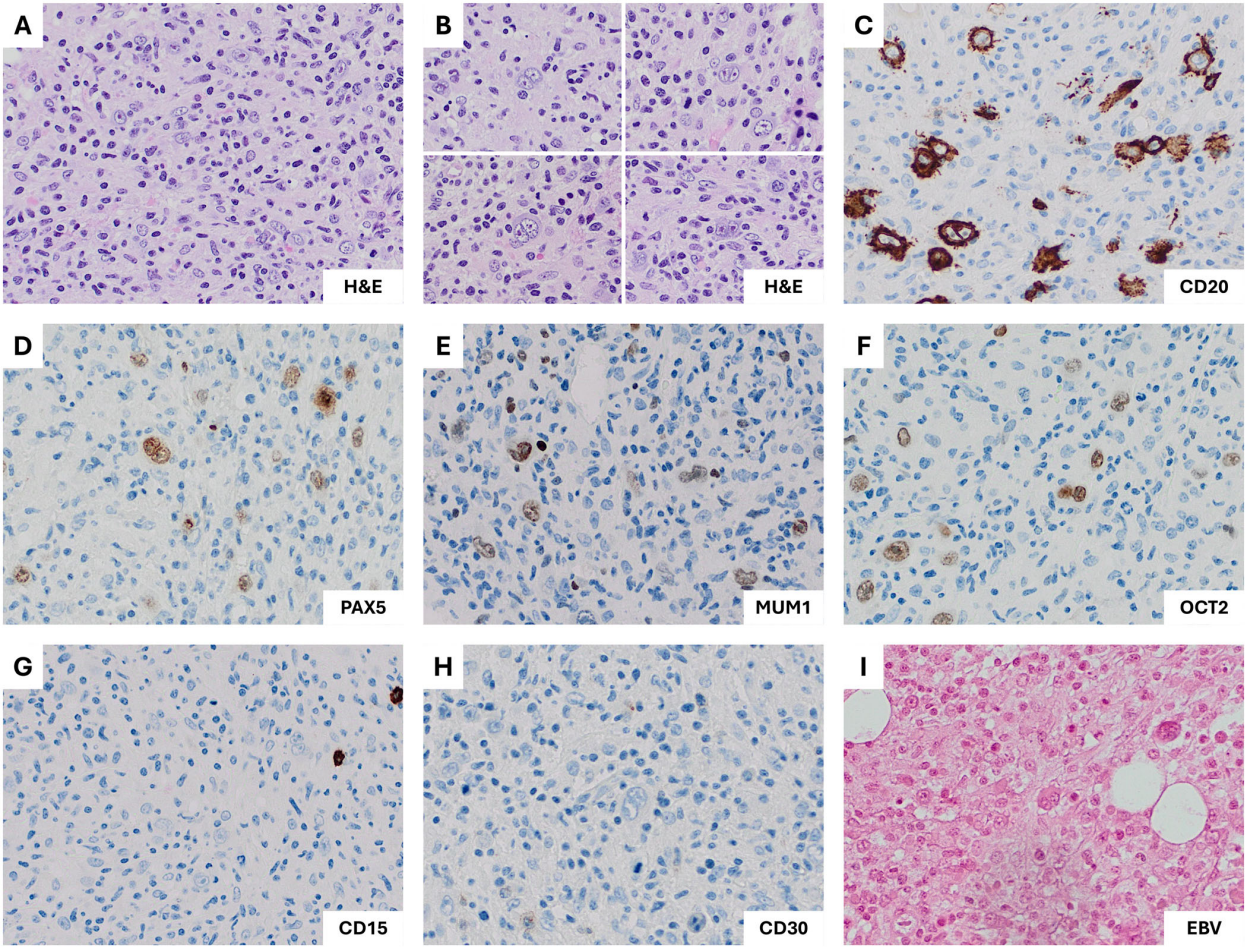
Bone marrow biopsy. (A, B) Haematoxylin and eosin staining (20× magnification) showing a polymorphous lymphohistiocytic infiltrate with large Hodgkin Reed–Sternberg‐like cells containing bilobed nuclei. (C–I) Immunohistochemistry (20× magnification) demonstrating that the atypical large cells are positive for CD20 (C), PAX5 (D), MUM1 (E) and OCT2 (F), and negative for CD15 (G), CD30 (H) and EBV‐encoded RNA (I).

HRS cells are the histological hallmark of classic Hodgkin lymphoma (cHL) and are large and multinucleated with prominent eosinophilic nucleoli, resulting in a characteristic ʻowl‐eyeʼ appearance. HRS cells are derived from mature B cells, but exhibit a unique immunophenotype (CD30+, CD15+, weak PAX5+, MUM1+ with weak/absent expression of other B cell markers and EBV positivity in a subset) reflecting downregulation of the B cell transcriptional program and constitutive activation of NF‐κB, JAK/STAT and immune evasion pathways [1]. Despite their defining association with cHL, HRS‐like cells may be seen in other lymphoproliferative disorders including THRLBCL and anaplastic large cell lymphoma [2, 3], underscoring the importance of comprehensive immunohistochemical profiling to establish an accurate diagnosis.

## Author Contributions

U.M., S.L. and K.L.C. analysed the data. M.A.A. provided clinical care. U.M. and K.L.C. wrote the manuscript with critical input from S.L. and M.A.A.

## Funding

The authors have nothing to report.

## Ethics Statement

Ethics approval was provided by the Human Research and Ethics Committee at the Peter MacCallum Cancer Centre (HREC reference PMC 03/90).

## Consent

The patient provided written informed consent.

## Conflicts of Interest

Mary Ann Anderson is an employee of the Walter and Eliza Hall Institute, which receives milestone payments in relation to venetoclax, to which she is entitled to a share. Mary Ann Anderson has received honoraria from AbbVie, AstraZeneca, Janssen, Lilly, Gilead, Novartis, Roche, Takeda, CSL and Specialised Therapeutics. The other authors declare no conflicts of interest.

## Data Availability

The data that support the findings of this study are available from the corresponding author upon reasonable request.
